# Regulation of JAK/STAT signal pathway by miR-21 in the pathogenesis of juvenile idiopathic arthritis

**DOI:** 10.1007/s12519-019-00268-w

**Published:** 2019-10-22

**Authors:** Hong-Wei Li, Hua-Song Zeng

**Affiliations:** 1 Department of Pediatric Allergy Immunology and Rheumatology, Guangzhou Women and Children Medical Center, Guangzhou 510623, China; 2 Department of Pediatric Allergy, Immunology and Rheumatology, Guangzhou Women and Children’s Medical Center, Guangzhou Medical University, 9 Jinsui Road, Guangzhou 510175, China

**Keywords:** JAK/STAT, Juvenile idiopathic arthritis, MiR-21, Signal transducer and activator of transcription 3 (STAT3)/p-STAT3

## Abstract

**Background:**

Overexpression of the components of the Janus kinase/signal transducer and activator of transcription (JAK/STAT) signalling pathway is the key factor of the pathogenic mechanisms underlying systemic juvenile idiopathic arthritis (sJIA). The study aims to investigate the association between miR-21 and the JAK/STAT signal pathway in JIA.

**Methods:**

Total RNA was extracted from peripheral blood mononuclear cells (PBMCs) in active JIA patients. The relative expressions of miR-21, STAT3 and suppressor of cytokine signalling 3 in PBMCs were measured by real-time polymerase chain reaction and their expressions were measured by western blotting and dual-luciferase reported assay. Rheumatoid arthritis fibroblast-like synovial cell (RASF) was stimulated to become to osteoclasts using macrophage colony-stimulating factor (M-CSF) and factors that can impact on their differentiation ability were identified through the transfection of LV3-miR-21. The expression of STAT3/p-STAT3 was measured by western blot, and the levels of interleukin (IL)-17A, p65, matrix metalloproteinases (MMP)-3, MMP-4 and receptor activator of nuclear factor-κB after the LV3-miR-21 transfection were tested by enzyme-linked immunosorbent assay. Finally, the miR-21 targeted *STAT3* gene was detected by the dual-luciferase reported assay.

**Results:**

The expression of miR-21 was significantly lower in JIA patients than in healthy control (*P* < 0.05). The level of STAT3 was increased in PBMCs of JIA group compared with control group (*P* < 0.05). Furthermore, the expression levels of miR-21 in sJIA and polyarticular JIA groups were negatively correlated with STAT3 (*r* = − 0.5854/*r* = − 0.6134, *P* < 0.05). The expression of STAT3 changed little in PBMCS after the stimulation of IL-6 and not in RASFs with transfection of LV3-miR-21. The expression of p-STAT3 decreased after the stimulation of IL-6 in RASFs transfected by LV3-miR-21 (*P* < 0.05). RASFs were induced into osteoclasts using M-CSF. The number of osteoclasts as determined by tartrate-resistant acid phosphatase staining was significantly lower in group miR-21 mimics as compared with the negative control group (*P* < 0.05).

**Conclusions:**

We showed that expression of miR-21 was significantly lower in JIA patients compared with healthy control. MiR-21 might affect the JAK/STAT signal pathway by suppressing the expression of STAT3 and phosphorylation of STAT3. MiR-21 could inhibit the production of osteoclasts induced from RASFs by M-CSF.

## Introduction

Juvenile idiopathic arthritis (JIA) is a chronic inflammatory disease which is characterized by persistent joint inflammation that manifests as joint pain and swelling while limiting the range of motion in the joints. It can lead to the short-term and long-term disability [1, 2]. This disease often presents with various clinical signs. However, there is usually non-specific laboratory evidence of inflammation at diagnosis and its pathogenesis is still ambiguous [2]. Therefore, JIA is difficult to diagnose early and its prognosis may be dependent on many factors. The current diagnosis of JIA relies mainly on clinical and radiographic findings, and may not provide correct diagnosis during the early stage of this disease. Thus, more sensitive and specific biomarkers are needed for an earlier diagnosis so that earlier treatment may improve the prognosis of these patients.

MicroRNAs (miRNAs) are non-coding RNAs, regulating gene expression of targeted mRNAs, which are implicated in the pathogenesis of autoimmune diseases [2]. MiRNAs can inhibit target gene function by binding combining to the 3′-untranslater region (3′-UTR) of the targeted genes involved in cell growth, proliferation, differentiation and the regulation of the cell cycle [3]. Altered expression of miRNAs has been described under various pathological conditions, including rheumatic and other autoimmune diseases [4]. With the help of microarray analysis and quantitative polymerase chain reaction (qPCR), more and more miRNAs were found to be related to arthritis such as miR-16, miR-155 [5], miR-146a [6], miR-223 and miR-132 [7]. Mature miR-146a and primary miR-146a/b were highly expressed in synovial tissue [8], and may reduce the expression of interleukin receptor associated kinase 1 [9]. Expression of miR-223 is increased in polyarthritis and has been correlated with matrix metalloproteinase-3 (MMP3) [10]. However, there are few studies about the target genes of miRNAs that might demonstrate their roles in immunological dysregulation and chronic inflammation. Also, there are few studies about miRNAs on different subtypes of JIA.

MiR-21 is a well-known microRNA which is involved in the development of a variety of diseases, such as cancer and inflammatory diseases [11]. MiR-21, also has an important role in Treg biology, promoting Th17 differentiation depending on signal transducers and activators of transcription 3 (STAT3) and is preferentially activated by interleukin-6 (IL-6) [12–14]. The level of miR-21 was significantly lower in rheumatoid arthritis patients and was accompanied by the increase of STAT3, as well as a decrease of STAT5/pSTAT5 protein and forkhead box P3 mRNA levels [15]. Another study showed that the high level of miR-21 could regulate the factor B cell lymphoma 6 (Bcl6), and then activate the expression of STAT3 [4, 5]. Zhou et al. found that knockout of miR-21 could inhibit the expression of STAT3 in vivo [6]. Moreover, there are also some studies suggesting that STAT3 could influence the expression of miR-21 [16]. From the above data, it seems that miR-21 and STAT3 have a close relationship. But whether miR-21 exerts its function via combining STAT3 directly and whether mutual regulation of miR-21 and STAT3 has an impact on the inflammatory factors downstream is still unclear. As JIA is an inflammatory disease, we may ask, can the expression of miR-21 have an influence on the Janus kinase/signal transducer and activator of transcription (JAK/STAT) signal pathway in this disorder? It is also important to ask, what is the role of miR-21 in JIA?

In our previous study [17], we have explored the relationship between miR-21 and JAK/STAT signal pathway in rheumatoid peripheral blood mononuclear cells (PBMCs). However, it still has not been identified if miR-21 can exert its function by combining STAT3 directly. In this study, the aim was to find the exact mechanism of miR-21 and STAT3 functioning in the chronic inflammation and osseous destruction of JIA using specific methods such as dual-luciferase reporter, mutant of STAT3 3′-UTR and western blot for detecting the downstream protein expression of STAT3.

## Methods

### Baseline collection

In our previous study [17], thirty-three active phase JIA patients, aged from 2 to 14 years, who were seen in the Rheumatology Department of Guangzhou Women and Children’s Medical Center, May 2017–July 2018, were included in this study. They belonged to two subgroups of JIA: systemic juvenile idiopathic arthritis (sJIA) (data were reported in a previous publication) [17] and polyarticular juvenile idiopathic arthritis (pJIA), conforming to the JIA classification of the 2001 International League of Associations for Rheumatology [18]. Twenty normal control patients were recruited from the Health Care Section of the hospital (the control data were reported in the previous publication) [17]. In this study, the patients had not started to use steroid or disease-modifying anti-rheumatic drugs after diagnosis and patients with other chronic diseases such as infections, hematopathy, and tumors were excluded. All the sampling were take in fasting condition. The study was performed based on an informed consent and was approved by the Ethics Committee of Guangzhou Women and Children’s Hospital, Guangzhou, Guangdong, China. Informed written consent was obtained from all individuals. The baseline characteristics are shown in Table 1.

**Table 1:** Comparison of baseline characteristic between patients with juvenile idiopathic arthritis and healthy control

Clinical characteristics	Case group (*n* = 33)		Control group (*n* = 20)	*P* value
	sJIA group (*n* = 20)	pJIA group (*n* = 13)		
Age (y), mean ± SD	7.36 ± 2.71	6.80 ± 2.23	6.56 ± 2.18	4.409
Sex (male:female)	13:7	8:5	14:6	0.853
Weight (kg), mean ± SD	13.45 ± 6.34	14.17 ± 5.47	12.67 ± 7.83	1.074
DAS28, mean ± SD	6.23 ± 2.34	6.17 ± 1.78	–	
MRI (synovitis/erosion), *n* (%)	16 (80)	13 (100)	–	
Anti-AKA positivity, *n* (%)	1 (5.0)	7 (53.3)	–	
Anti-CCP positivity, *n* (%)	2 (10.0)	10 (76.9)	–	
RF-IgG positivity, *n* (%)	15 (75.0)	9 (69.2)	–	
FER (ng/mL), mean ± SD	1079.47 ± 900.87	138.12 ± 45.14	–	
CRP (mg/L), mean ± SD	98.61 ± 63.80	89.74 ± 34.85	1.76 ± 0.84	0.001
ESR (mm/h), mean ± SD	53.12 ± 26.59	37.00 ± 19.32	2.57 ± 1.15	0.001

*sJIA* systemic juvenile idiopathic arthritis, *pJIA* polyarticular juvenile idiopathic arthritis, *CCP* cyclic citrullinated peptide, *AKA* anti-keratin antibody, *FER* ferritin, *RF-IgG* rheumatoid factor IgG, *CRP* C-reactive protein, *ESR* erythrocyte sedimentation rate, *MRI* magnetic resonance imaging, *SD* standard deviation

### Reagents

Ficoll-Hypaque PLUS solution and phosphate-buffered saline (PBS) were purchased from GE Healthcare Life Sciences (Logan, UT, USA); the RNA extraction kit (9112; Takara Biotechnology Co. Ltd. Dalian, China), reverse transcription kit, RNAiso (9753A), miR-X iRNA First Strand Synthesis kit (638515) and SYBR Premix EX Taq (Tli R NaseH Plus; RR420A) kits were purchased from Takara Biotechnology Co, Ltd. The cell culture medium and materials were OptiMEM medium (31985-062; Gibco, USA), DMEM (11965-092; Gbico, USA), fetal bovine serum (FBS) (1168944; Gibco, USA), Trypsin (Gibco, USA), penicillin–streptomycin (Pen/Strep) (15140-122; Gibco, USA). LV3-miR-21 (Gene Pharma, 293 T cell, COA-020 3.0 × 10^9^/mL, TAGCTTATCAGACTGATGTTGA), LV3-negative control (NC) (Gene Pharma, 293 T cell, C22AZ 2.0 × 10^9^/mL, TTCTCCGAACGTGTCACGTTTC).

### Isolation of total RNA from PBMCs and cDNA synthesis

3 mL blood was collected from JIA patients and healthy volunteers, in sodium ethylene diamine tetraacetate anticoagulant tubes and diluted in. 4 mL of PBS equal volumes of Ficoll Paque PLUS solution and diluted blood were added into 15 mL centrifuge tubes and centrifuged at 160 × *g* for 20 minutes at 18–21 °C. The cell pellet was then collected, resuspended in PBS, and centrifuged at 160 × *g* for 20 minutes for 2 cycles. 1 mL RNAiso and 200–300 mL chloroform (Sigma-Aldrich, St. Louis, MO, USA) was added to the cell pellet and the samples were incubated for 5 minutes at 25–28 °C and centrifuged at 12,000 × *g* for 15 minutes at 4 °C. Approximately 400 µL supernatant was obtained, to which 1 mL cold isopropanol (Sigma-Aldrich) was added, mixed and incubated at 4 °C for 10 minutes, prior to being centrifuged at 12,000 × *g* for 10 minutes at 4 °C. The supernatant was then discarded, and 10 mL 75% ethanol prepared with diethylpyrocarbonate (DEPC) (Sigma-Aldrich) water was added (ethanol:DEPC 3:1), followed by centrifugation at 7500 × *g* for 5 minutes at 4 °C. Finally, 20 µL DEPC water was added to the samples, and the absorbance was measured using a spectrophotometer (Thermo NanoDrop 2000; Thermo Fisher Scientifc, Inc, Waltham, MA, USA); reverse transcription of RNA into cDNA was done using miR-X iRNA First Strand Synthesis kit. cDNA were stored in − 20 °C. All manipulation was proceeded according to the manufacturer’s protocol. Rheumatoid arthritis fibroblast-like synovial cells (RASFs) were from biopsy of JIA patients and stored in − 80 °C.

### Detecting the expression of miR-21 and its associated mRNAs in PBMCs by quantitative real-time PCR (qRT-PCR)

The mRNA expression levels of specific genes were quantified by qRT-PCR. The primers of miR-21 (5′-GTCGTATCCAGTGCAGGGTCCGAGGTATTCGCACTGGATACGACTCAACA-3′) and U6 (5′-CTCAACTGGTGTCGTGGAGTCGGCAATTCAGTTGAGAAAAATATG-3′) were synthesized by TAKARA.

The primers of *STAT3* (F: 5′-GCCAGAGAGCCAGGAGCA-3′, R: 5′-ACACAGATAAACTTGGTCTTCAGGTATG-3′), suppressor of cytokine signaling 3 (*SOCS3*) (F: 5′-CAGCTCCAAGAGCGAGTACC-3′, R: 5′-TGACGCTGAGCGTGAAGAAG-3′), *β-actin* (F: 5′-GAGCTACGAGCTGCCTGACG-3′, R: 5′-GTAGTTTCGTGGATGCCACAG-3′) were synthesized by Beijing Genomics Institute.

Expression of MMP-3, MMP-4, receptor activator of nuclear factor-κB ligand (RANKL) and NF-κb was detected after the LV3-miR-21 transfected RASFs were treated with IL-6 for 1, 2, 4 and 8 hours, respectively. The sequences of the primers were as follows:

*MMP-3* (F: 5′-CACTCACAGACCTGACTCGGTT-3′, R: 5′-AAGCAGGATCACAGTTGGCTGG-3′),

*MMP-4* (F: 5′-CCTGACAAAGCACGGCAAGAAC-3′, R: 5′-CCAGCACCTTGGAACTTCTGTC-3′),

*RANKL* (F: 5′-GCCTTTCAAGGAGCTGTGCAAAA-3′, R: 5′-GAGCAAAAGGCTGAGCTTCAAGC-3′),

*NF-*κ*b* (F: 5′-ACGATCTGTTTCCCCTCATC-3′, R: 5′-GCTTCTCTCCCCAGGAATACTGCC-3′).

### Cell culture and transfection of LV3-miR-21 into RASFs

RASFs were thawed from storage and subcultured for 2–3 times to 80–90% confluence. Cells were inoculated into 6-well plates according to a cell density of 6 × 10^5^ cells/well. Serum-free medium was replaced the next day and multiplicity of infection (MOI) of 50 of virus was added to the culture, incubated for 2 hours and then culture media was replaced by fresh complete medium. After 48 hours, the cells were collected and passaged in 1:2. After adherence, the cells were screened by adding puromycin at a concentration of 10 µg/mL. The solution was changed every 2–3 days (puromycin complete medium with 10 µg/mL). One week later, the medium was replaced with puromycin complete medium of 2 µg/mL. The RASFs were suspended in a concentration of 2 × 10^5^ cells/mL in DMEM (11965-092, Gibco) supplemented with 10% heat-inactivated FBS and 1% Pen/Strep in 6-well plates for 24 hours at 37 °C. When the cell density reached 70–80% confluence, the cells were transfected with LV3-miR-21 lentivirus vector (Gene Pharma) and LV3-NC according to the manufacturer’s instructions. Cells were then harvested and subjected to qRT-PCR analysis of miR-21 expression.

### Cell viability assay

RASFs were seeded into 96-well plates and incubated with treating with IL-6 of different concentrations (0, 1, 10, 100, and 150 ng/mL) for 0, 0.5, 1, 2, and 4 hours, respectively. Subsequently, 100 μL fresh medium was added to cells containing 10 μL Cell Counting Kit-8 solution (Dojindo Laboratories, Tokyo, Japan) and incubated for 1 hour (37 °C, 5% CO_2_). Absorbance at 450 nM was measured using a microplate reader (Cytation5, BioTech, USA).

### Inducing the differentiation of RASF-derived osteoclasts and identifying factors that can impact on their differential ability

RASFs were inoculated on 6-well plates at a concentration of 1 × 10^5^ cells per well. After culturing overnight, the medium was discarded and replaced with 2 mL of fresh medium. Macrophage colony-stimulating factor (M-CSF) was added to a final concentration of 30 ng/mL. The cells were placed in a 37 °C, 5% CO_2_ incubator for 5 days. The differentiation into osteoclasts was identified by tartrate-resistant acid phosphatase (TRAP) staining. Then the RASF-derived osteoclasts were divided into five groups: mock group, LV-miR-21 group, LV-miR-21 + phosphorylation inhibitors, LV-miR-21 + phosphorylation inhibitor mock and blank group. To verify the differentiation ability of these groups, cells were treated with IL-6 (100 ng/mL) for 1, 2, 4 and 8 hours, respectively, and the cells were stained with TRAP.

### Dual-luciferase reported assay

293 T cells (1 × 10^4^) were seeded in 500 μL medium into wells of a 12-well plate. After 18–24 hours of incubation, cells were transfected for 1–2 hours with 1 μg reporter plasmid (STAT3 3′-UTR and STAT3 Mut 3′-UTR) (Transgen, Beijing, China) using 250 μL Optim and 3 μL Lipofectamine 2000 according to the manufacturer’s instructions. After transfection, the medium was replaced with 500 μL medium with 10% FBS and incubated for another 6 hours. Dual-luciferase reporter assay was performed 48–72 hours after the transfection. Cells were harvested by adding 200 μL passive lysis buffer at room temperature. After centrifuging, 100 μL supernatant was assayed for luciferase detection using a microplate luminometer (Cytation5, BioTech, USA). Transcription factor-binding sites in promoter region of STAT3 with miR-21 was predicted with bioinformatics analysis.

### Western blot analysis

RASFs (*n* = 5) were collected and dissolved using 200 μL RIPA buffer (Beibo, China) with 2 mM protease inhibitors sodium orthovanadate (Beibo, China) and 2 μL phosphatase inhibitors (Beibo, China) for 30 min on ice and then centrifuged at 10,000 rpm for 30 minutes at 4 °C. After, the total protein concentrations were measured using the BCA method (Biyuntian, China). Equal amounts (30 μg) of protein were separated by 10% sodium dodecyl sulfate denatured polyacrylamide gel and transferred onto poly (vinylidene fluoride) membranes in bovine serum albumin in Tris-based saline with Tween 20 for 1 hour at room temperature. Membranes were incubated with rabbit antihuman antibodies at the recommended dilution [STAT3 at a dilution of 1:100 (Abcam, USA), p-STAT3 (Tyr705) at 1:10,000 (Abcam, USA), SOCS3 at 1:10,000 (Abcam, USA), GAPDH at 1:5000 (Abcam, USA)] overnight at 4 °C. After washing in Tris-based saline with Tween 20, the membranes were further incubated with a secondary anti-rabbit antibody (1:10,000) for 2 hours. Enhanced chemiluminescence solution was added onto the membranes, and protein expression was quantified using the Laboratory Work Image Acquisition and Analysis Software (UVP, Upland, CA). GAPDH was used as a loading control.

### Enzyme-linked immunosorbent assay (ELISA)

Levels of IL-17A, p65, MMP-3, MMP-4 and RANKL were measured by ELISA according to the manufacturer’s protocols (USCN Life Science Inc., Wuhan, China; Kamiya Biomedical Co., KT-58997, Tukwila, WA, USA).

### Statistical analysis

SPSS 11.0 software was used and all data were expressed as mean (SD). Differences between the two groups were assessed using Kruskal–Wallis test or Student’s *t* test, whereas the correlation of multiple groups was analyzed using Pearson's correlation coefficient. The Student’s *t* test was employed for pairwise comparisons between two groups in baseline, and two-way ANOVA was used for intergroup comparisons. *P* < 0.05 indicated statistically significant.

## Results

### Baseline

For JIA [sJIA (*n* = 20), pJIA (*n* = 13)] patients and healthy controls, their demographic and clinical characteristics were recorded, including age, sex, rheumatoid factors, anti-citrullinated protein antibody, C-reactive protein, erythrocyte sedimentation rate, magnetic resonance imaging and ferritin (FER) (Table 1). No significant difference was found in age, sex or weight between two groups (*P* > 0.05). There were significant differences in anti-keratin antibody (AKA), cyclic citrullinated peptide (CCP) and FER between the sJIA and pJIA groups (*P* < 0.05). Positive for RF IgG of sJIA was highly 75% (15/20) (Table 1), 25% (5/20) positive in second review. The positive percentage of AKA and CCP was higher in the pJIA group compared with the sJIA group, while the mean level of FER increased in the sJIA group. The characteristics of baseline coincided with the study.

### Expression of miR-21, STAT3 and SOCS3 mRNA of PBMCs and their correlations in JIA

**Fig. 1:**
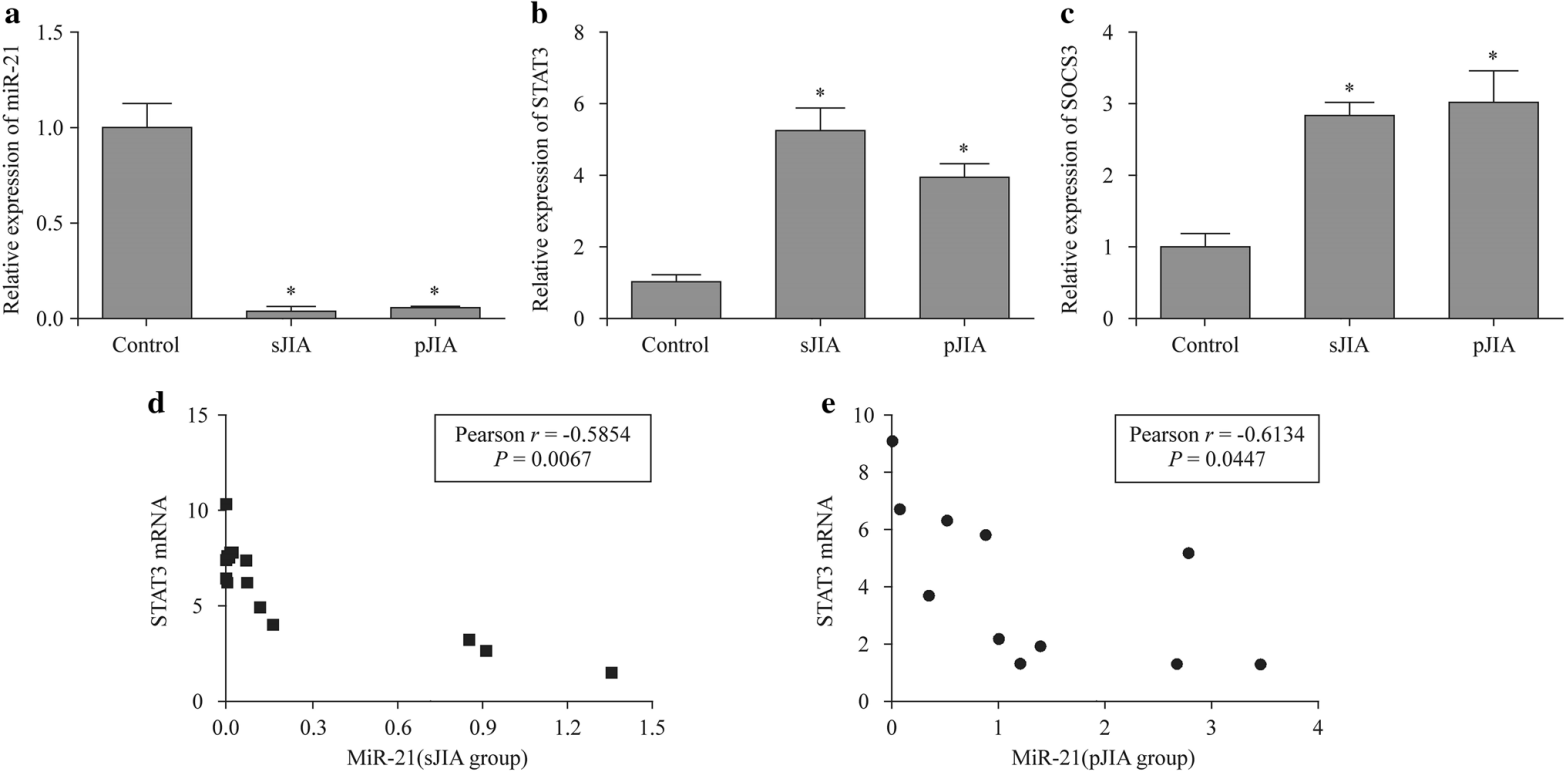
Expression of miR-21, STAT3 and SOCS3 mRNA of PBMCs and their correlation in JIA.* sJIA* systemic juvenile idiopathic arthritis,* pJIA* polyarticular juvenile idiopathic arthritis,* STAT3* signal transducers and activators of transcription 3,* SOCS3* suppressor of cytokine signaling 3,* PBMCs* peripheral blood mononuclear cells. * *P* < 0.05

In our previous study [19], we decided to compare the expression of miR-21 in PBMC and from JIA [sJIA (*n* = 20), pJIA (*n* = 13)] patients and healthy controls. Indeed, as shown in Fig. 1a, expression of miR-21 was significantly lower in JIA patients than the healthy controls (*P* < 0.05), and the miR-21 expression of group sJIA and group pJIA was 7.7 times and 6.49 times lower than the control groups (Table 2). However, there was no significant difference of miR-21 expression between group sJIA and pJIA. Moreover, level of *STAT3*, *SOCS3*, *IL-6*, tumor necrosis factor-α (*TNF-α*) gene increased in PBMCs of JIA group compared with control group (*P* < 0.05) (Fig. 1b, c). We also found that the expression level of miR-21 in sJIA and pJIA group was correlated negatively with STAT3 (*r* = − 0.5854/*r* = − 0.6134, *P* < 0.05) (Fig. 1d, e).

**Table 2:** Expression of miR-21 and STAT3/SOCS3 in sJIA and pJIA group

Group	Case group		Control (*n* = 20)	*Z*/*P* value
	sJIA (*n* = 20)	pJIA (*n* = 13)		
MiR-21	7.70 (7.00, 8.50)	6.49 (6.00, 7.00)	1.02 (0.64, 1.45)	2.615/0.015, 2.334/0.029
STAT3	6.24 (2.81, 7.54)	3.97 (1.81,5.75)	1.00 (0.56, 1.29)	4.869/0.001, 3.804/0.008
TNF-α	3.03 (2.07, 3.80)	3.42 (2.46, 4.68)	1.09 (0.93, 2.01)	3.356/0.002, 5.146/0.001
IL-6	4.75 (3.59, 6.32)	3.52 (2.31, 7.51)	1.87 (1.23, 2.54)	3.082/0.006, 2.388/0.036
SOCS3	2.54 (1.77, 4.00)	3.57 (1.95, 3.83)	0.86 (0.21, 1.20)	3.344/0.003, 4.783/0.001

*sJIA* systemic juvenile idiopathic arthritis, *pJIA* polyarticular juvenile idiopathic arthritis, *STAT3* signal transducers and activators of transcription 3, *SOCS3* suppressor of cytokine signaling 3, *TNF-α* tumor necrosis factor-α, *IL-6* interleukin-6

### Transfection of LV3-miR-21 suppressed p-STAT3/STAT3 protein expression

**Fig. 2:**
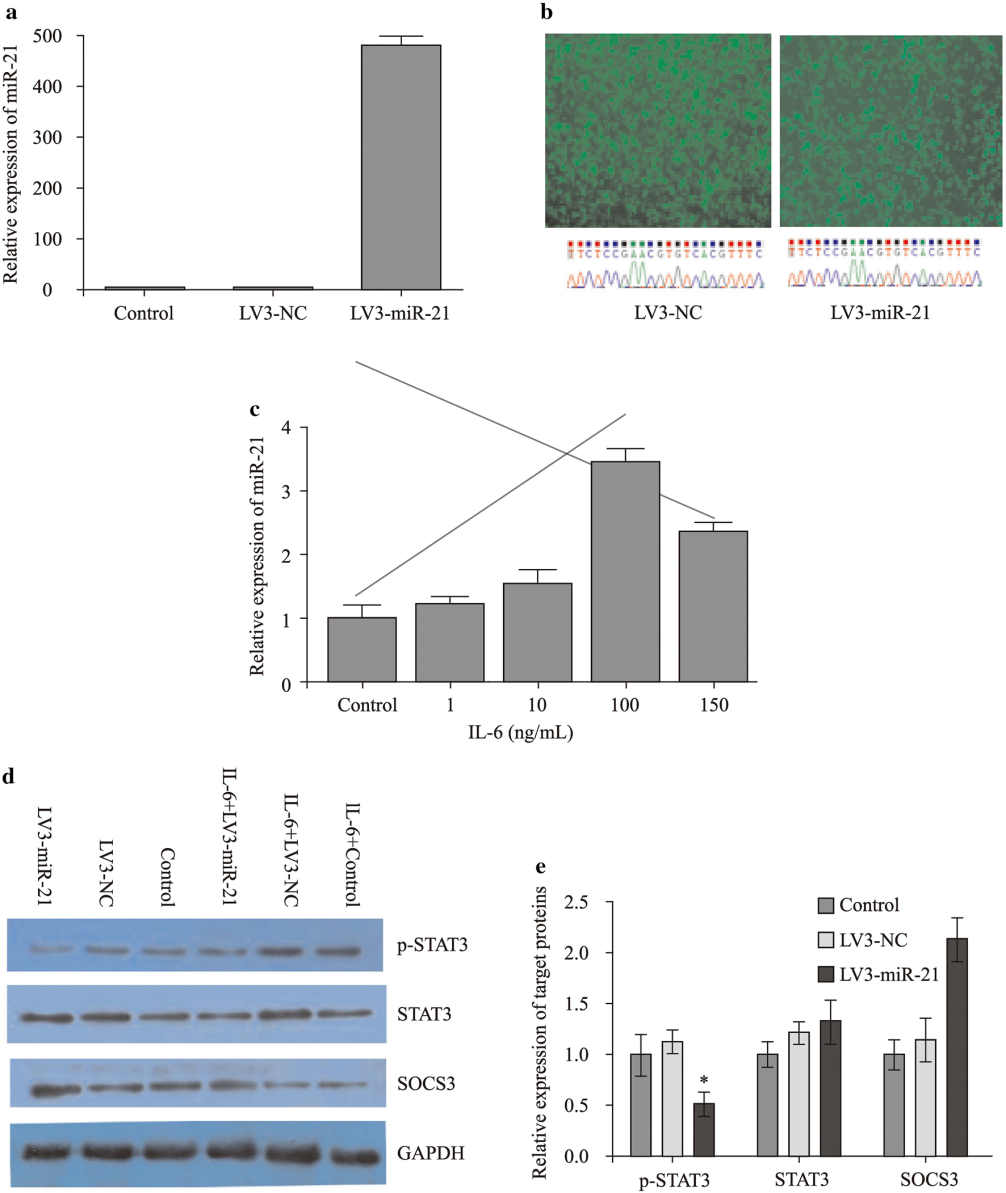

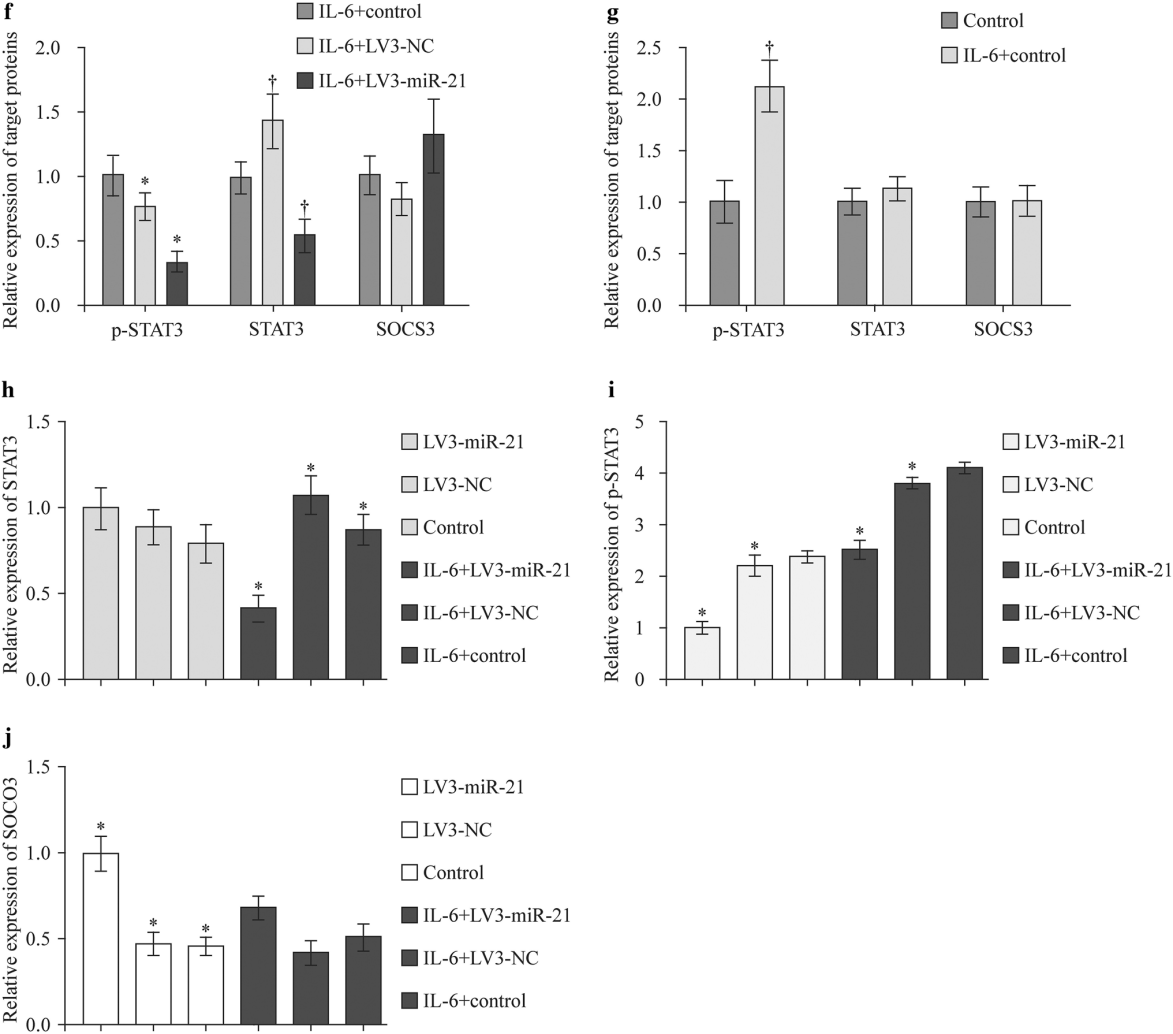
Transfection of LV3-miR-21 suppressed p-STAT3/STAT3 protein expression: **a**, **b** The titer of miR-21 in RASFs after LV3-miR-21 transfection; **c** OD value of RASFs with the simulation of IL-6; **d**–**j** The expression of STAT3/p-STAT3/SOCS3 in RASFs after transfection of LV3-miR-21 with the simulation of IL-6.* STAT3* signal transducers and activators of transcription 3,* SOCS3* suppressor of cytokine signaling 3,* IL-6* interleukin-6,* RASF* rheumatoid arthritis fibroblast-like synovial cell,* NC* negative control, * *P* < 0.05, † *P* < 0.05

To investigate the connection between miR-21 and STAT3, LV3-miR-21 and LV3-NC were transformed into RASFs that could express p-STAT3/STAT3 proteins. After a 5-day transfection, the expression levels of miR-21 dramatically increased after LV3-miR-21 transfection (Fig. 2a, b). Then RASFs were stimulated with IL-6 (0, 10, 100, 150 µg/mL) for 4 hours (Fig. 2c). The upregulation of miR-21 in the RASFs was associated with suppression of phosphorylation of STAT3 and upregulation of SOCS3. However, level of STAT3 protein did not change by overexpressing miR-21 (Fig. 2d, e). Moreover, we found that LV3-miR-21 could suppress the protein translation of STAT3 in RASFs stimulated with IL-6 (*P* < 0.05), but no change had been found in the IL-6 absent group (Fig. 2d–g). A typical presentation was shown that the STAT3 greatly increased with the presence of IL-6 in RASFs transfected with LV3-NC compared with control group while it decreased in LV3-miR-21 group (Fig. 2h). While the p-STAT3 decreased in LV3-miR-21 group in absent of IL-6 and stimulation of IL-6 (Fig. 2i). The level of SOCS3 decreased in RASFs with the stimulation of IL-6 (*P* < 0.05) (Fig. 2j).

### Enhancing miR-21 or silencing STAT3 could suppress the IL-6 induced regulation of targeted genes

**Fig. 3:**
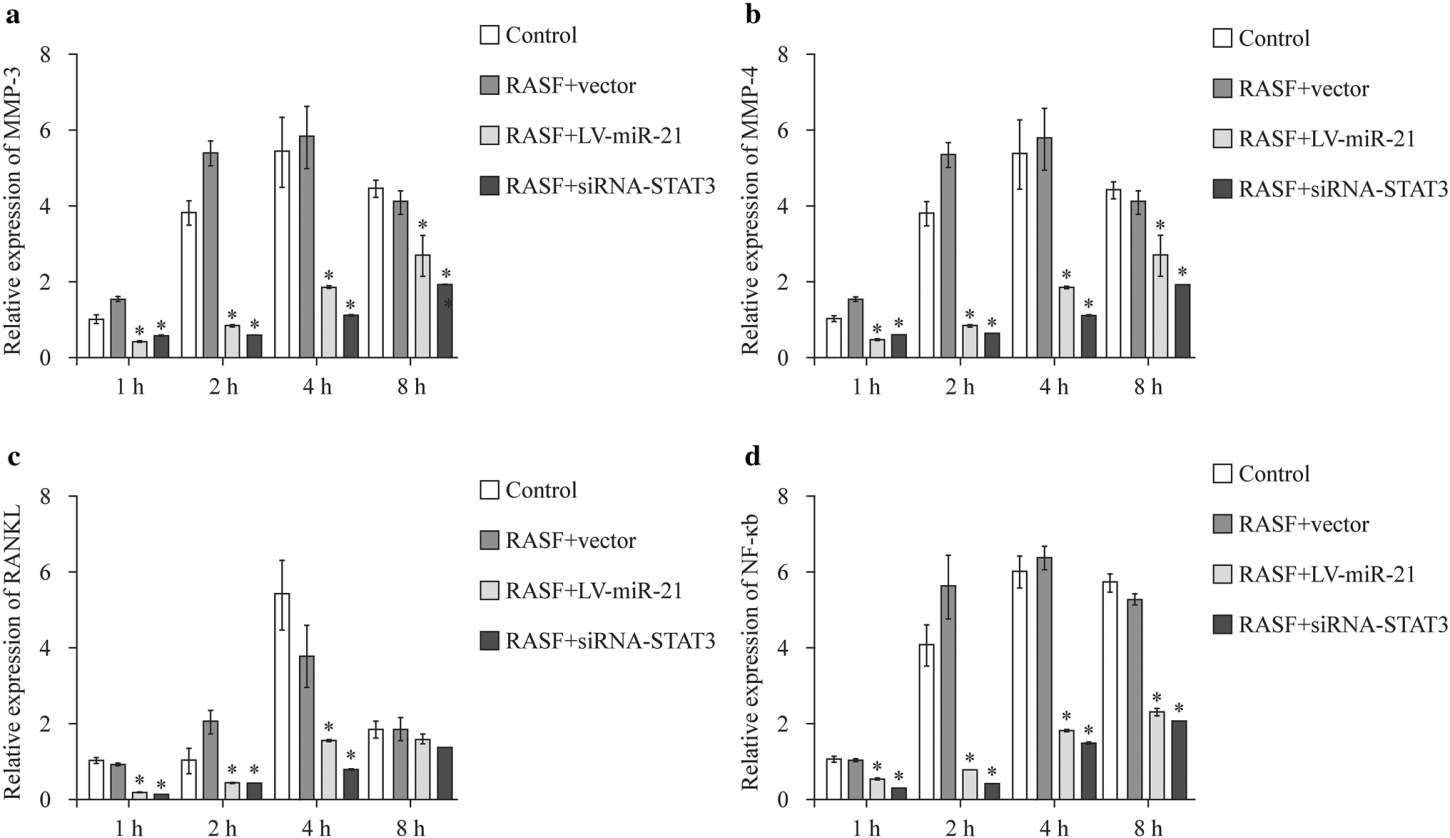
Enhancing miR-21 or silencing STAT3 could suppress the IL-6 induced regulation of targeted genes.* STAT3* signal transducers and activators of transcription 3,* IL-6* interleukin-6,* RASF* rheumatoid arthritis fibroblast-like synovial cell,* MMP* matrix metalloproteinase,* RANKL* receptor activator of nuclear factor-κB ligand,* NF-κb* nuclear factor-κB

RASF cells were divided into four groups: control, RASF + vector, RASF + LV-miR-21 and RASF + siRNA-STAT3. They were treated with IL-6 (100 ng/mL) for 0, 1, 4, 8 hours. Then expression of *MMP-3*, *MMP-4*, *RANKL* and *NF-κb* was detected by qRT-PCR. It showed that the expression of these genes could be upregulated by treating IL-6, the optimal treating time was 4 hours (Fig. 3). However, increasing the expression of miR-21 with LV-miR-21 or silencing *STAT3* with siRNA-STAT3 could effectively suppress this IL-6-induced effect (Fig. 3).

### Enhancing miR-21 or silencing STAT3 could suppress the IL-6-induced regulation of targeted proteins

**Fig. 4:**
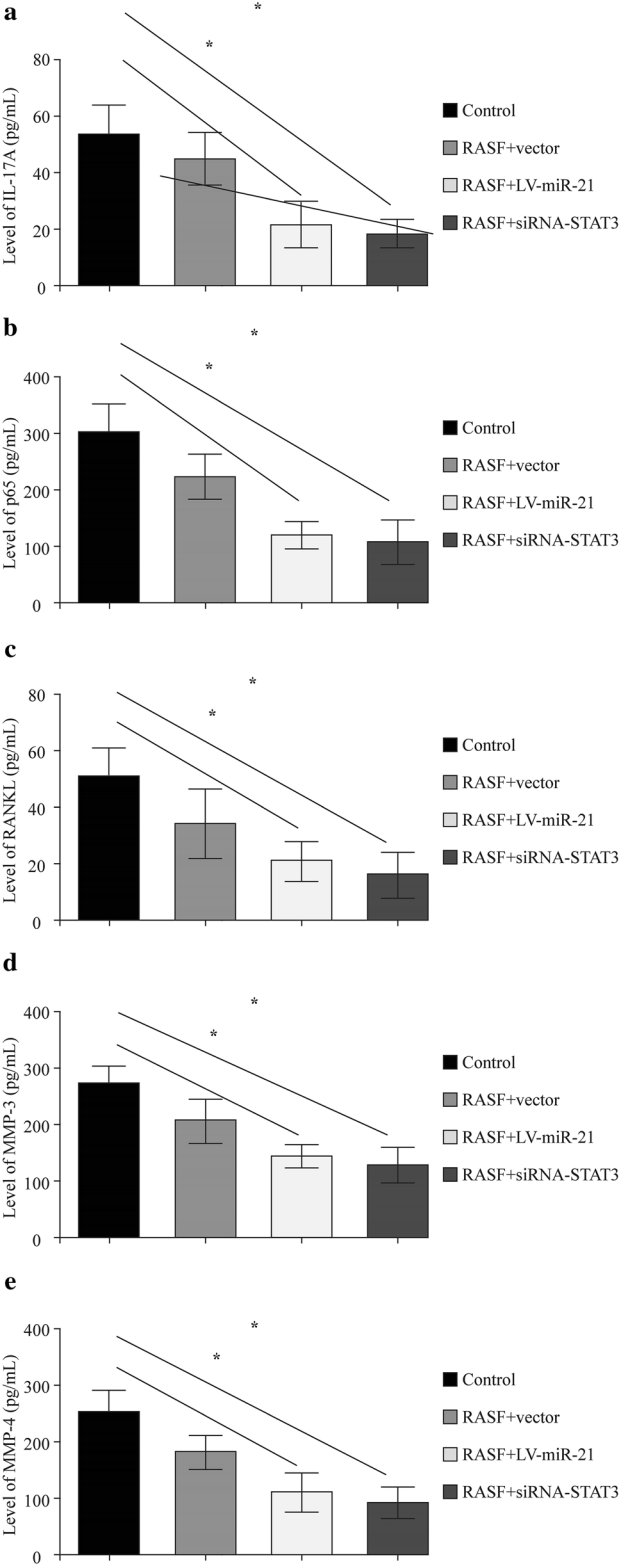
Enhancing miR-21 or silencing STAT3 could suppress the expression of p65 pathway.* STAT3* signal transducers and activators of transcription 3,* IL* interleukin,* RASF* rheumatoid arthritis fibroblast-like synovial cell,* MMP* matrix metalloproteinase,* RANKL* receptor activator of nuclear factor-κB ligand

Four groups (control, RASF + vector, RASF + LV-miR-21 and RASF + siRNA-STAT3) of RASF cells were treated with IL-6 (100 ng/mL) for 4 hours, then the protein level of IL-17A (Fig. 4a), p65 (Fig. 4b), RANKL (Fig. 4c), MMP-3 (Fig. 4d) and MMP-4 (Fig. 4e) was measured by ELISA assays. The result showed that the levels of these proteins were significantly lower in groups of RASF + miR-21 and RSAF + siRNA-STAT3 (Fig. 4).

### MiR-21 exerts its functions via targeting *STAT3* gene directly

**Fig. 5:**
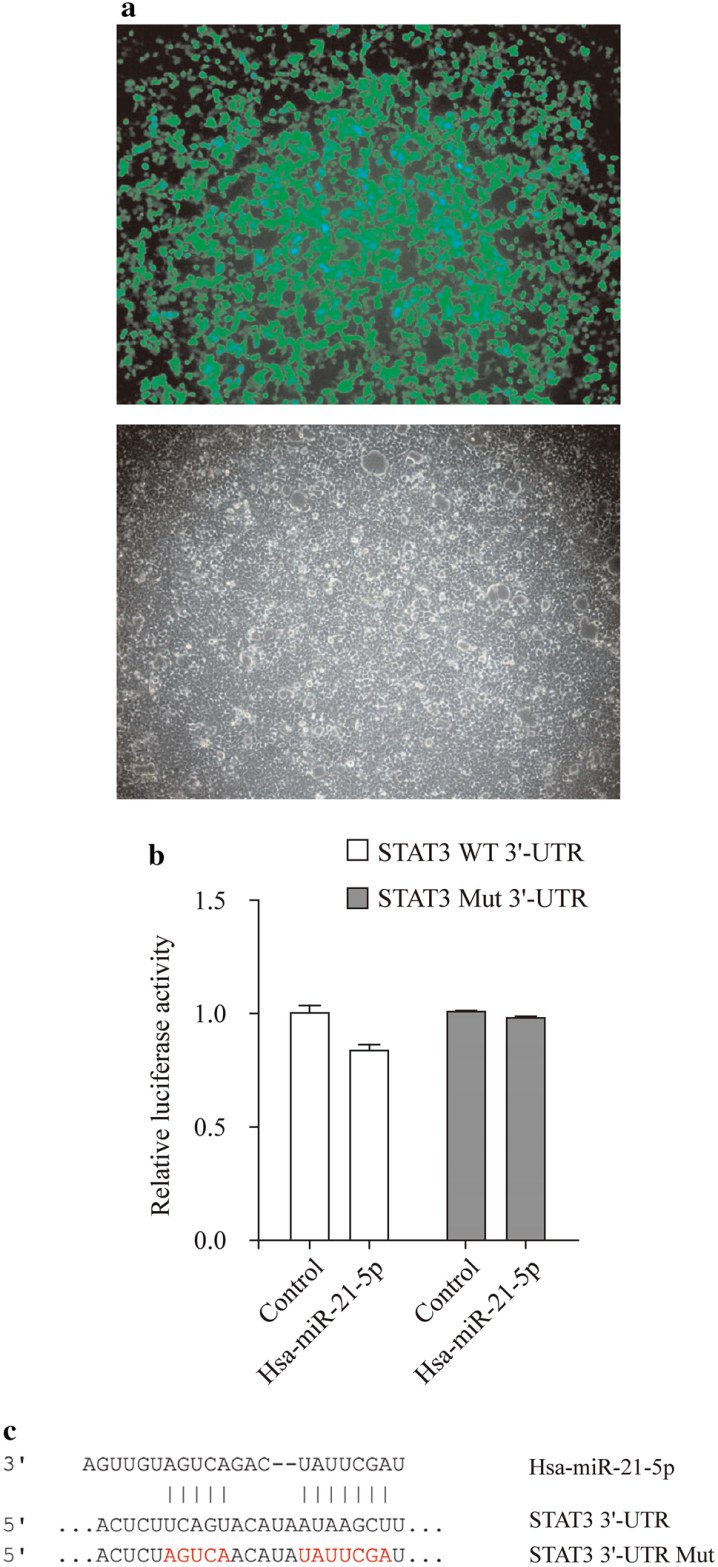
The luciferase activities of the RASFs with STAT3-3′UTR dropped after being co-transfected with hsa-miR-21-5p mimics.* STAT3* signal transducers and activators of transcription 3,* RASF* rheumatoid arthritis fibroblast-like synovial cell,* 3′-UTR 3′*-untranslater region

To further investigate whether miR-21 is a functional target of STAT3, a dual-luciferase reporter assay was performed. We predict and design hsa-miR-21-5p target 2040 to 2046 on STAT3 3′-UTR with bioinformatics analysis. The results showed that the luciferase activities of the cells with STAT3-3′-UTR dropped after being co-transfected with hsa-miR-21-5p mimics, comparing with the control group using miR-21 mimic NC instead. The luciferase activity of the mimic group was 83.468% of the NC group, which meant that hsa-miR-21-5p mimics could slightly inhibit the expression of gene *STAT3*. As a comparison, 293 T cells with mutant STAT3-3′-UTR were also transfected with hsa-miR-21-5p mimics and miR-21 mimics NC. However, the decrease of luciferase activities of the mimic group was 97.523% of the NC group (Fig. 5).

### MiR-21 could inhibit the production of osteoclasts induced from RASFs by macrophage colony-stimulating factor (M-CSF)

**Fig. 6:**
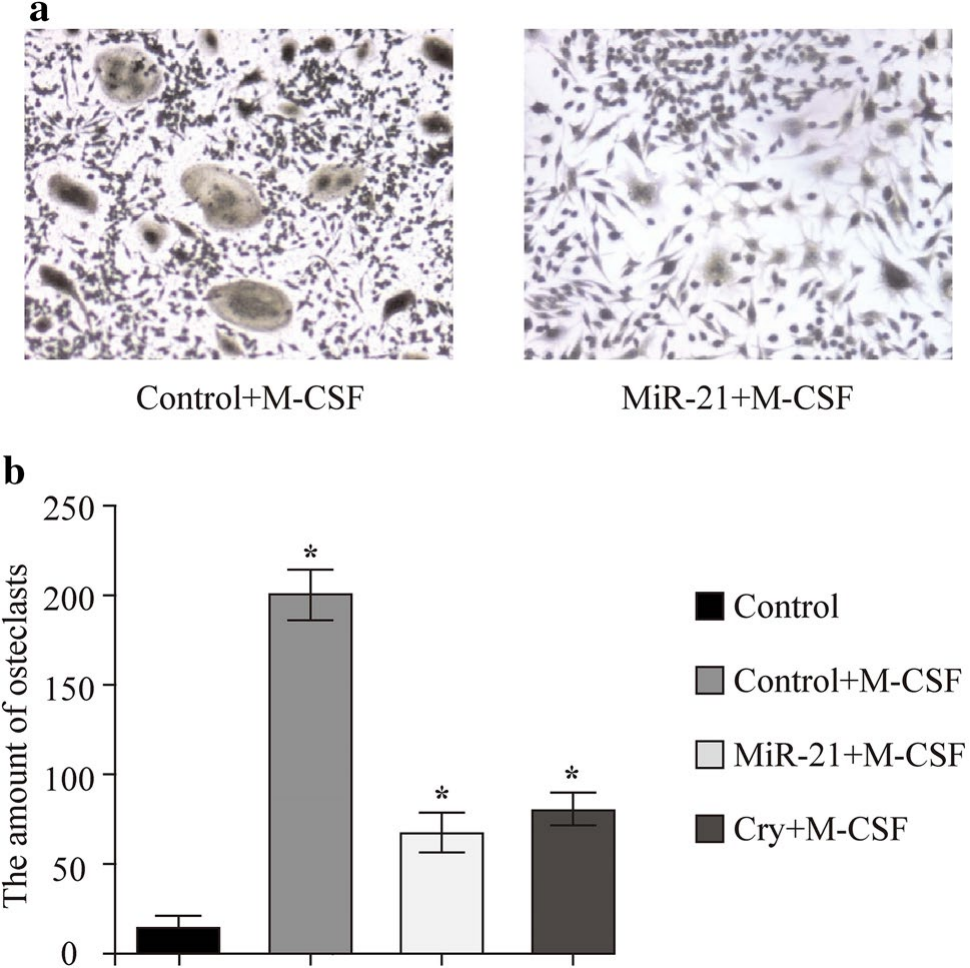
M-CSF was used for inducing the RASFs to osteoclasts.* RASF* rheumatoid arthritis fibroblast-like synovial cell,* M-CSF* macrophage colony-stimulating factor. * P < 0.05

CSF was used for inducing the RASFs to develop into osteoclasts. Based on TRAP staining, the amounts of osteoclasts in group miR-21 mimics were significantly lower than the NC group (*P* < 0.05) (Fig. 6a). Moreover, the decrease of cell amounts could also be found in the NC group which was transfecting with miR-21 mimics after the treatment of inhibitor of STAT3 phosphorylation (cryptotanshinone) with no difference between the group miR-21 mimics and group STAT3 phosphorylation inhibition (Fig. 6b). Therefore, miR-21 has a similar function as STAT3 inhibitor on inhibiting the production of osteoclasts.

## Discussion

JIA is a heterogeneous condition with a variable outcome and considerable ongoing disease burden [18]. MiR-21 has been described as an oncogenic miRNA exhibiting anti-apoptotic activity in various carcinomas and inflammations. It can, not only regulate the level the inflammatory factor, such as TNF-α and IL-10 secretion [19], but also be induced by many pro-inflammatory stimuli, such as pathogen-associated molecular patterns and damage-associated molecular patterns. This suggests that miR-21 may be an important factor for the resolution of inflammation with the negative regulation of the inflammation process [20]. As shown in Fig. 1a, expression of miR-21 was significantly lower in JIA patients than healthy controls (*P* < 0.05). It may be helpful to suppress the chronic inflammation such as JIA by increasing the level of miR-21. Dong et al. [21] showed that IL-6 activated prostate cancer pathway through the targeted function of miR-21. IL-6 was increased in both sJIA and pJIA, and miR-21 levels in PBMCs were not different between these 2 subtypes of JIA.

Many studies showed that miR-21 was related to different inflammatory signal pathways. The miR-21 can regulate the proliferation of dendritic cells [22] and B cells [23] by the LPS-TLR4-NFκB pathway; and increased miR-21 expression in the monocyte lineage cells, may be related to the PMA-mediated activation of activator protein 1 [24].

The JAK–STAT pathway mediates important biological mechanisms, including inflammation, cell proliferation and antiviral activity, and is stimulated by receptor binding of cytokines, such as IL-6 [25, 26]. After IL-6 stimulation, the target cell launches an intracellular signal transduction cascade via the JAK/STAT pathway, leading to the phosphorylation of STAT3 [27–29]. Indeed we had demonstrated that STAT3 were abnormally upregulated in the PBMCs of JIA patients as shown in Fig. 1b. Furthermore, we had found that the expression of miR-21 was negatively correlated with STAT3 (Fig. 1d, e). Therefore, whether *STAT3* is the target gene for miR-21 is an important issue that needs resolution.

Our study showed that the expression level of miR-21 was negatively correlated with STAT3 in PBMCs of JIA (*r* = − 0.5854/*r* = − 0.6134, *P* < 0.05) (Fig. 1d, e). We inferred that STAT3 might suppress the expression of miR-21 in PBMCs of JIA. But which role did miR-21 play in JAK/STAT signal pathway? Recent studies showed that STAT3 might correlate with IL-6-induced miR-21 [30]. Clinical data strongly indicated that IL-6 and IL-6R may play a key role in the induction and progression of JIA and its complications [31].

Data in Fig. 2h shows hardly any difference in STAT-3 whether IL-6 is added or not. The only difference is the addition of LV3-miR-21 inhibits STAT-3 expression in RASFs stimulated with IL-6, but LV3-miR21 transfection had no effect on STAT-3 expression when IL-6 is not added to the culture system. So we inferred that over-expression of miR-21 can decrease the level of STAT3 only during inflammation (as indicated by the presence of IL-6).

However, whether STAT3 is the target gene of miR-21 remains unknown. In our study, we showed that miR-21 could exert its functions by combining with the *STAT3* gene directly by dual-luciferase assay. Hsa-miR-21-5p mimics (target by 2040 to 2046 on STAT3 3′-UTR) could slightly inhibit the expression of gene *STAT3*. As a comparison, 293 T cells with mutant STAT3-3′-UTR were also transfected with hsa-miR-21-5p mimics and miR-21 mimics NC. It can be concluded that gene miR-21 could inhibit pro-inflammatory activities and bone destruction by binding to its target gene *STAT3*.

Our findings showed that miR-21 could inhibit the phosphorylation of the target protein STAT3 (Fig. 2i). The p-STAT3 greatly increased with the presence of IL-6 in RASFs transfected with LV3-NC and in control group while decreased in LV3-miR-21 group, shown in Fig. 2i. A study showed the Bcl6 is an important regulator of Th cells, and could inhibit Th2 inflammation. At present, it is thought that miR-21 is the target of Bcl6, and is able to downregulate Bcl6 expression and activate STAT3 [32, 33]. Dong et al. [15] found that the phosphorylation of STAT3 can be actively sustained after the knockout of miR-21 in a transplanted tumor. In tumor model, downregulation of miR-21 was associated with downregulation of STAT3 [33]. This is in contrast to data in this paper, which is upregulation of miR-21 was associated with downregulation of STAT3. So one will have to be very careful in drawing parallel between the malignant diseases and inflammatory diseases, and we deduce that miR-21 may inhibit production of p-STAT3-the phosphorylation of STAT3. In our study, it was shown that miR-21 downregulated the expression of p-STAT3/STAT3 and the inflammation seen in JIA is due to low miR-21. It was also identified that miR-21 could regulate the expression of some key inflammatory factors such as IL-17, p65, MMP-3, MMP-4, and RANKL by inhibiting the STAT3 phosphorylation which finally leads to the chronic inflammation and bone destruction of JIA. RANKL is a member of inflammation and bone destruction of JIA.

The decreased expression of miR-21 in JIA might be related to the differences in pathogenesis. Understanding the exact role of miR-21 in regulating JAK/STAT signal will be valuable in the development of miR-21-targeted diagnosis and therapy strategy of JIA.
